# Factors associated with discharge destination from acute care after acquired brain injury in Ontario, Canada

**DOI:** 10.1186/1471-2377-12-16

**Published:** 2012-03-24

**Authors:** Amy Y Chen, Brandon Zagorski, Daria Parsons, Rika Vander Laan, Vincy Chan, Angela Colantonio

**Affiliations:** 1 Toronto Rehabilitation Institute, 550 University Ave, Toronto, ON M5G 2A2, Canada; 2 Department of Occupational Science and Occupational Therapy, Faculty of Medicine, University of Toronto, Rehabilitation Sciences Building, 160-500 University Ave, Toronto, ON M5G 1V7, Canada; 3 Department of Health Policy, Management and Evaluation, Faculty of Medicine, University of Toronto, Health Sciences Building, 155 College Street, Suite 425, Toronto, ON M5T 3M6, Canada; 4 Undergraduate Medical Education, School of Medicine, Queen's University, 80 Barrie Street, Kingston, ON K7L 3N6, Canada; 5 Dalla Lana School of Public Health, Faculty of Medicine, University of Toronto, Health Sciences Building, 155 College Street, Toronto, ON M5T 3M6, Canada

## Abstract

**Background:**

The aim of this paper is to examine factors associated with discharge destination after acquired brain injury in a publicly insured population using the Anderson Behavioral Model as a framework.

**Methods:**

We utilized a retrospective cohort design. Inpatient data from provincial acute care records from fiscal years 2003/4 to 2006/7 with a diagnostic code of traumatic brain injury (TBI) and non-traumatic brain injury (nTBI) in Ontario, Canada were obtained for the study. Using multinomial logistic regression models, we examined predisposing, need and enabling factors from inpatient records in relation to major discharge outcomes such as discharge to home, inpatient rehabilitation and other institutionalized care.

**Results:**

Multinomial logistic regression revealed that need factors were strongly correlated with discharge destinations overall. Higher scores on the Charlson Comorbidity Index were associated with discharge to other institutionalized care in the nTBI population. Length of stay and special care days were identified as markers for severity and were both strongly positively correlated with discharge to other institutionalized care and inpatient rehabilitation, compared to discharge home, in both nTBI and TBI populations. Injury by motor vehicle collisions was found to be positively correlated with discharge to inpatient rehabilitation and other institutionalized care for patients with TBI. Controlling for need factors, rural location was associated with discharge to home versus inpatient rehabilitation.

**Conclusions:**

These findings show that need factors (Charlson Comorbidity Index, length of stay, and number of special care days) are most significant in terms of discharge destination. However, there is evidence that other factors such as rural location and access to supplemental insurance (e.g., through motor vehicle insurance) may influence discharge destination outcomes as well. These findings should be considered in creating more equitable access to healthcare services across the continuum of care.

## Background

Acquired brain injury (ABI), which can be traumatic and non-traumatic, is a leading cause of death and disability in North America and worldwide [1,2]. There are enormous consequences for the person affected, their families and the health care system [1,3]. As such, it is important to understand how the characteristics of individuals with ABI affect their trajectory across the health care system, as this will ensure better preparation in acute care, inform public policy, and improve program planning. Furthermore, identifying factors that are correlated with discharge destination may assist in improving resource planning at the facility level [4].

The aim of this study is to identify factors that influence discharge destination by traumatic (TBI) and non-traumatic (nTBI) brain injury using a comprehensive database of hospitalization records in Ontario and selecting ABI cases based on the International Classification of Diseases version 10 [5]. Previous research has found longer lengths of stay [6], worse Glasgow coma scale (GCS) score [6], age [6], sex [6], race/ethnicity [6-9], source of payment [6], scores on adaptability test, and length of post-traumatic amnesia [10] to be factors associated with discharge to inpatient rehabilitation. In addition, being over the age of 65 was also found to be associated with being discharged to a nursing home facility [10]. However most of these studies have limited sampling and rely on registries, which can miss a large fraction of TBI patients. To date, there is a paucity of comprehensive data on discharge destination after ABI from a population based perspective in Canada.

Using the Anderson Behavioral Model, we identified factors associated with the use of services after acute care admission for brain injury from administrative databases. This model identifies predisposing factors (demographic characteristics, social structure, and beliefs), need factors (indicators of perceived and diagnosed severity of health condition), and enabling factors (family financial situation and community resources) as strong influences on health care usage [11,12]. Population based administrative data from publicly insured jurisdictions can provide insight into the trajectory of individuals with ABI through the public health care system. Furthermore, identifying factors associated with different discharge outcomes of persons with brain injury is valuable to practitioners and program managers. This information will enable better case planning and management across the system; patients and their family can also use this information in forming their expectations of their hospitalization and the possible outcomes of their hospital stay.

## Methods

### Study design and population

This was a retrospective cohort study in an ABI population in Ontario, Canada from April 1, 2003 to October 31, 2006. Patients' records were captured using Ontario hospital discharge abstracts in the Discharge Abstract Database (DAD), collected by the Canadian Institute of Health Information, and provided to us by the Ontario Ministry of Health and Long-Term Care (MOHLTC). Ontario has a universal publicly funded health care system, with mandatory reporting of emergency department visits, hospitalizations, and inpatient rehabilitation. ABI cases discharged alive were identified in the DAD by the presence of an ICD-10 code for TBI and nTBI in any diagnosis position (up to 25). nTBI included brain infections, brain tumours, anoxia, metabolic encephalopathies, toxic effects, and vascular insults excluding stroke (See Table 1). As we were not interested in studying recurrent conditions, only the first admission in the study period was examined and admissions in 2003 were excluded due to an insufficient look back period. Individuals with a stroke diagnosis in any position were excluded from the TBI cohort and a diagnosis of stroke in the most responsible diagnosis position (i.e., the condition most responsible for the length of stay) in the nTBI cohort was also excluded. Since TBI and nTBI patients have different demographics and utilize healthcare differently, they were analyzed as separate cohorts.

**Table 1:** ICD-10 Definitions used for TBI and NTBI

TBI Codes	
Fracture of the skull	S02 [.0, .1, .7-.9]

Intracranial injury	S06 [.0-.6, .8, .9]

Sequelae of injury	T90 [.2, .5, .8, .9], T96, T97, T98.2

nTBI Codes *	

Brain infections	A81.1, A83.0, A83.2, A87 [.0-.2, .8, .9], B00.4, B01.0, B01.1, B02.0, B05.0, B37.5, G00 [.0-.3, .8, .9], G01.0, G02 [.0, .1, .8], G03 [.0-.2, .8, .9], G04 [.0, .8, .9], G05 [.0-.2, .8], G06 [.0-.2], G93.0

Encephalopathy	E10.0, E11 [.0, .1], E13 [.0, .1], E14 [.0, .1] E15, F07.2

Toxic effects	T51 [.0, .1, .2, .3, .8-.9], T56 [.0, .1, .4, .5, .8, .9], T58

Anoxia	G93.1, T75.1, T71

Vascular insults	I62.0, I62.9

Brain neoplasms	C70 [.0, .1, .9], C71 [.0-.9], C79.3, D32.0, D33 [.0-.3], D42.0, D43 [.0-.4, .7, .9]

*Patients were excluded with stroke codes (I60, I61, I63, and I64) in the most responsible position

### Anderson behavioral model variables

Variables abstracted were selected based on the framework set by the Anderson Behavioral Model. Predisposing factors identified in the DAD included age and sex at discharge. Need factors, which indicate the severity of the patient's conditions, included the Charlson Comorbidity Index, length of stay in acute care, and number of special care days (i.e., sum of all days in all intensive care units). The Charlson Comorbidity Index has been widely accepted as a useful tool for measuring comorbidity disease status and has been shown to have a consistent correlation to in-hospital mortality [13]. Enabling factors identified included whether the TBI was a result of a motor vehicle collision (MVC), which served as a proxy of supplemental insurance to pay for associated healthcare costs. In addition, rural residences were determined by individual postal code and designated as being rural by the Canadian Postal Service.

### Outcome variables

Discharge destination from acute care was determined by the 'discharge disposition' and the 'institutional to' type variables (i.e., a code identifying the level of care of the facility to where the patient was transferred) in DAD. Three main categories were created for the purpose of analysis:

1) Home: with or without support including senior's lodge, attendant care, home care, meals on wheels, homemaking and supportive housing;

2) Inpatient rehabilitation

3) Other institutionalized care: other inpatient hospital care (other acute, sub-acute, psychiatric, cancer centre/agency and pediatric hospital), long term care facilities (personal care homes, auxiliary care, nursing homes, extended care, homes for the aged, seniors' homes), and other facilities (palliative care/hospice, addiction treatment center, etc.).

### Statistical analysis

Frequency distributions and measures of central tendency were generated for all the variables and also stratified by TBI and nTBI patient groups. Within each discharge destination, the number of individuals was also examined for each variable considered. Predictor variables were categorized according to standard intervals (Charlson Comorbidity Index), percentiles (length of stay), and by standard cutoffs (special care days). Multinomial logistic regression was used to calculate odds ratios and 95% confidence intervals for the likelihood to be discharged to either inpatient rehabilitation or other institutionalized care relative to being discharge home. Full models containing all Anderson Behavioral variables are presented regardless of statistical significance. Multicollinearity was evaluated using variance inflation factor > 4.

### Privacy and ethics

This study received ethics approval from the Toronto Rehabilitation Institute Research Ethics Board. All investigators and staff involved in the study signed confidentiality agreements, and analyses were conducted with de-identified data. Data were stored on a secure server and analysis was conducted on a password protected computer located on premises with additional security access. No data tables with less than five counts were displayed.

## Results

Tables 2 and 3 present the predisposing, need and enabling factors for TBI and nTBI. The majority of TBI and nTBI patients were males, however, the gender distribution was more equitable in the nTBI population. Compared to TBI patients, nTBIs were older, had a higher Charlson Comorbidity Index score, and increased length of hospital stay. The distribution of special care days was similar between TBI and nTBI, with approximately 25% requiring one or more special care days. A similar percentage of TBI and nTBI patients were living in rural areas (~20%) and among TBI patients, 20.9% were due to a MVC.

**Table 2:** Discharge Destination of TBI Population (N = 10,443)

Characteristic	TBI Overall [n (Col %)]	Home [n (Col %, Row %)]	Rehabilitation [n (Col%, Row %)]	Other [n (Col %, Row %)]
**Total**	10,443 (100)	7,804 (100, 74.7)	1,019 (100, 9.8)	1,620 (100, 15.5)

Sex *				

Male	6,883 (65.9)	5,245 (67.2, 76.2)	676 (66.3, 9.8)	962 (59.4, 14.0)

Female	3,559 (34.1)	2,559 (32.8, 71.9)	343 (33.7, 9.6)	657 (40.6, 18.5)

Age at Discharge (Years) *				

< 18	2,181 (20.9)	2,098 (26.9, 96.2)	17 (1.7, 0.8)	66 (4.1, 3.0)

18-24	1,060 (10.2)	850 (10.9, 80.2)	129 (12.7, 12.2)	81 (5.0, 7.6)

24-34	847 (8.1)	649 (8.3, 76.6)	104 (10.2, 12.3)	94 (5.8, 11.1)

35-44	1,028 (9.8)	795 (10.2, 77.3)	114 (11.2, 11.1)	119 (7.4, 11.6)

45-54	1,092 (10.5)	801 (10.3, 73.4)	148 (14.5, 13.6)	143 (8.8, 13.1)

55-64	1,036 (9.9)	760 (9.7, 73.4)	121 (11.9, 11.7)	155 (9.6, 15.0)

65-74	1,009 (9.7)	696 (8.9, 69.0)	112 (11.0, 11.1)	201 (12.4, 19.9)

75+	2,187 (20.9)	1,153 (14.8, 52.7)	274 (26.9, 12.5)	760 (46.9, 34.8)

**Charlson Comorbidity Index**				

0-1	9,685 (92.7)	7,436 (95.3, 76.8)	899 (88.2, 9.3)	1,350 (83.3, 13.9)

2-3	624 (6.0)	310 (4.0, 49.7)	106 (10.4, 17.0)	208 (12.8, 33.3)

≥ 4	134 (1.3)	58 (0.7, 43.3)	14 (1.4, 10.4)	62 (3.8, 46.3)

**Length of Stay (Days)**				

1-2	2,415 (23.1)	2,262 (29.0, 93.7)	4 (0.4, 0.2)	149 (9.2, 6.2)

3-5	2,778 (26.6)	2,488 (31.9, 89.6)	47 (4.6, 1.7)	243 (15.0, 8.7)

6-11	2,347 (22.5)	1,775 (22.7, 75.6)	195 (19.1, 8.3)	377 (23.3, 16.1)

12+	2,903 (27.8)	1,279 (16.4, 44.1)	773 (75.9, 26.6)	851 (52.5, 29.3)

**Special Care Days**				

None	7,249 (69.4)	5,847 (74.9, 80.7)	386 (37.9, 5.3)	1,016 (62.7, 14.0)

1-2	1,381 (13.2)	1,114 (14.3, 80.7)	115 (11.3, 8.3)	152 (9.4, 11.0)

3-5	752 (7.2)	487 (6.2, 64.8)	128 (12.6, 17.0)	137 (8.5, 18.2)

6-11	519 (5.0)	225 (2.9, 43.4)	156 (15.3, 30.1)	138 (8.5, 26.6)

12+	542 (5.2)	131 (1.7, 24.2)	234 (23.0, 43.2)	177 (10.9, 32.7)

**Motor Vehicle Collision**				

No	8,260 (79.1)	6,270 (80.3, 75.9)	670 (65.8, 8.1)	1,320 (81.5, 16.0)

Yes	2,183 (20.9)	1,534 (19.7, 70.3)	349 (34.3, 16.0)	300 (18.5, 13.7)

**Rural**				

No	8,340 (79.9)	6,162 (79.0, 73.9)	872 (85.6, 10.5)	1,306 (80.6, 15.7)

Yes	2,103 (20.1)	1,642 (21.0, 78.1)	147 (14.4, 7.0)	314 (19.4, 14.9)

Note: * Missing values were excluded and thus, column percentages may not add up to 100%

**Table 3:** Discharge Destination of nTBI Population (N = 27,223)

Characteristic	NTBI Overall [n (Col %)]	Home [n (Col %, Row %)]	Rehabilitation [n (Col %, Row %)]	Other [n (%Col %, Row %)]
**Total**	27,223 (100)	18,769 (100, 68.9)	2,392 (100, 8.8)	6,062 (100, 22.3)

Sex *				

Male	14,240 (52.3)	9,973 (53.1, 70.0)	1,276 (53.3, 9.0)	2,991 (49.3, 21.0)

Female	12,973 (47.7)	8,790 (46.8, 67.8)	1,116 (46.7, 8.6)	3,067 (50.6, 23.6)

Age at Discharge (Years) *				

< 18	2,111 (7.8)	1,904 (10.1, 90.2)	8 (0.3, 0.4)	199 (3.3, 9.4)

18-24	751 (2.8)	647 (3.5, 86.2)	38 (1.6, 5.1)	66 (1.1, 8.8)

24-34	1,298 (4.8)	1,115 (5.9, 85.9)	55 (2.3, 4.2)	128 (2.1, 9.9)

35-44	2,249 (8.3)	1,828 (9.7, 81.3)	153 (6.4, 6.8)	268 (4.4, 11.9)

45-54	3,412 (12.5)	2,715 (14.5, 79.6)	258 (10.8, 7.6)	439 (7.2, 12.9)

55-64	4,284 (15.7)	3,216 (17.7, 75.1)	387 (16.2, 9.0)	681 (11.2, 15.9)

65-74	5,112 (18.8)	3,338 (17.8, 65.3)	558 (23.3, 10.9)	1,216 (20.1, 23.8)

75+	7,976 (29.3)	3,983 (21.2, 49.9)	935 (39.1, 11.7)	3,058 (50.5, 38.3)

**Charlson Comorbidity Index**				

0-1	14,091 (51.8)	10,217 (54.4, 72.5)	1,250 (52.3, 8.9)	2,624 (43.3, 18.6)

2-3	6,700 (24.6)	4,091 (21.8, 61.1)	773 (32.3, 11.5)	1,836 (30.3, 27.4)

≥ 4	6,432 (23.6)	4,461 (23.8, 69.4)	369 (15.4, 5.7)	1,602 (26.4, 24.9)

**Length of Stay (Days)**				

1-2	2,509 (9.2)	2,001 (10.7, 79.8)	19 (0.8, 0.8)	489 (8.1, 19.5)

3-5	6,157 (22.6)	5,294 (28.2, 86.0)	145 (6.1, 2.4)	718 (11.8, 11.7)

6-11	7,591 (27.9)	5,900 (31.4, 77.7)	501 (20.9, 6.6)	1,190 (19.6, 15.7)

12+	10,966 (40.3)	5,574 (29.7, 50.8)	1,727 (72.2, 15.7)	3,665 (60.5, 33.4)

**Special Care Days**				

None	19,306 (70.9)	13,523 (72.1, 70.0)	1,398 (58.4, 7.2)	4,385 (72.3, 22.7)

1-2	3,779 (13.9)	3,009 (16.0, 79.6)	278 (11.6, 7.4)	492 (8.1, 13.0)

3-5	1,820 (6.7)	1,194 (6.4, 65.6)	230 (9.6, 12.6)	396 (6.5, 21.8)

6-11	1,142 (4.2)	589 (3.1, 51.6)	209 (8.7, 18.3)	344 (5.7, 30.1)

12+	1,176 (4.3)	454 (2.4, 38.6)	277 (11.6, 23.6)	445 (7.3, 37.8)

**Rural**				

No	22,746 (83.6)	15,636 (83.3, 68.7)	2,091 (87.4, 9.2)	5,019 (82.8, 22.1)

Yes	4,477 (16.5)	3,133 (16.7, 70.0)	301 (12.6, 6.7)	1,043 (17.2, 23.3)

Note: * Missing values were excluded and thus, column percentages may not add up to 100%

Table 2 shows the characteristics of the TBI population by discharge destination. The majority of individuals were discharged home (74.7%), followed by other institutionalized care (15.5%), and to inpatient rehabilitation (9.8%). As the age groups increased, the percentage discharged home decreased and the percentage to inpatient rehabilitation and 'other' increased. Similarly, as the Charlson Comorbidity Index score, length of stay in acute care, and the number of special care days increased, the percentage of patients discharged home decreased while the percentage discharged to inpatient rehabilitation and 'other' increased (see Table 2 for specific percentages). Among patients who were discharged home, 20% were involved in a MVC and 21% were living in a rural area. Similar percentages were found among patients discharged to 'other'. However, among those discharged to inpatient rehabilitation, 34% were involved in a MVC and 14% were living in a rural area.

Table 3 shows the discharge destinations of the nTBI population. The majority of individuals were discharged home (68.9%), followed by discharge to other institutionalized care (22.3%), and to inpatient rehabilitation (8.8%). As the age groups increased, the percentage discharged home decreased and the percentage to inpatient rehabilitation and 'other' increased. Specifically, among patients who were discharged home, 39% were older adults (aged 65 years and older) while the majority of patients who were discharged to inpatient rehabilitation (62.3%) and 'other' (60.6%) were older adults. Similar to TBI patients, as the Charlson Comorbidity Index, length of stay in acute care, and the number of special care days increased, the percentage discharged home decreased and the percentage to inpatient rehabilitation and to 'other' increased (see Table 3 for specific percentages). Among patients discharged home and to 'other', 17% were living in a rural area while 13% of those discharged to inpatient rehabilitation were living in a rural area.

Multinomial logistic regression revealed that predisposing, need, and enabling factors were significantly associated with discharge to inpatient rehabilitation compared to home among TBI patients. Specifically, compared to patients between the ages of 35 and 44, those under the age of 18 were significantly less likely to be discharged to inpatient rehabilitation (OR = 0.09) while patients aged 75 years and older were significantly more likely to be discharged to this destination (OR = 2.01). With increasing length of stay and number of special care days, there was a linear relationship in the odds of being discharged to inpatient rehabilitation. MVC also significantly increased the odds of discharge to inpatient rehabilitation (OR = 1.67) while living in a rural area significantly decreased the odds of discharge to this destination (OR = 0.75). Patients with a Charlson Comorbidity Index score of 2 to 3 were 1.32 times as likely as patients with a score of less than 1 to be discharged to inpatient rehabilitation compared to home. Gender was not significantly associated with discharge to inpatient rehabilitation compared to home (see Table 4).

**Table 4:** Multinomial Logistic Regression for TBI and Predisposing, Need and Enabling Characteristics

	Characteristic	Adjusted ORs (95% CI)	
		
		Rehabilitation vs. Home	Other vs. Home
**Predisposing Factors**	**Sex**		
	
	Male	1.00	1.00
	
	Female	1.02 (0.87-1.20)	1.02 (0.92-1.14)
	
	**Age**		
	
	< 18	0.09 (0.05-0.15)	0.22 (0.17-0.29)
	
	18-24	1.01 (0.73-1.38)	0.65 (0.51-0.85)
	
	25-34	1.02 (0.73-1.41)	0.94 (0.73-1.21)
	
	35-44	1.00	1.00
	
	45-54	1.32 (0.98-1.78)	1.35 (1.07-1.70)
	
	55-64	1.21 (0.89-1.65)	1.76 (1.41-2.21)
	
	65-74	1.22 (0.89-1.67)	2.73 (2.19-3.40)
	
	75+	2.01 (1.52-2.65)	7.63 (6.26-9.30)

**Need Factors**	**Charlson Comorbidity Index**		
	
	0-1	1.00	1.00
	
	2-3	1.32 (1.02-1.71)	1.93 (1.62-2.31)
	
	≥ 4	0.81 (0.44-1.49)	3.65 (2.61-5.10)
	
	**Length of Stay (Percentile)**		
	
	< 25^th^	1.00	1.00
	
	25-50^th^	6.63 (2.38-18.45)	0.42 (0.37-0.49)
	
	50-75^th^	29.72 (11.00-80.33)	0.44 (0.38-0.51)
	
	75-90^th^	98.96 (36.62-267.44)	0.71 (0.60-0.85)
	
	90^th^+	168.00 (61.59-458.25)	0.96 (0.77-1.20)
	
	**Special Care Days**		
	
	None	1.00	1.00
	
	1-2	1.77 (1.39-2.25)	2.18 (1.90-2.51)
	
	3-5	2.19 (1.72-2.80)	2.88 (2.40-3.45)
	
	6-11	3.41 (2.63-4.42)	5.42 (4.34-6.77)
	
	12+	4.90 (3.69-6.52)	6.95 (5.30-9.12)

**Enabling Factors**	**Motor Vehicle Collision**		
	
	No	1.00	1.00
	
	Yes	1.67 (1.39-2.00)	1.37 (1.20-1.57)
	
	**Rural**		
	
	No	1.00	1.00
	
	Yes	0.75 (0.61-0.92)	0.98 (0.87-1.12)

Predisposing, need, and enabling factors were also significantly associated with discharge to other institutionalized care compared to home among TBI patients. With patient age, Charlson Comorbidity Index score, length of stay, and special care days, there was a linear relationship in the odds of being discharged to 'other'. Patients involved in a MVC were 1.37 times as likely as patients who were not involved in a MVC to be discharged to 'other' compared to home (see Table 4).

Table 5 presents the multinomial logistic regression for nTBI patients. Predisposing, need, and enabling factors were all significantly associated with discharge to inpatient rehabilitation compared to home. Specifically, with patient age, length of stay in acute care, and number of special care days, there was a linear relationship with the odds of discharge to inpatient rehabilitation. Also, patients with a Charlson Comorbidity Index score of four or more (OR = 0.43) and those who were living in a rural area (OR = 0.78) were significantly less likely to be discharged to inpatient rehabilitation. Gender was not a significant predictor of discharge to inpatient rehabilitation.

**Table 5:** Multinomial Logistic Regression for nTBI and Predisposing, Need and Enabling Characteristics

	Characteristics	Adjusted ORs (95% CI)	
		
		Rehabilitation vs. Home	Other vs. Home
**Predisposing Factors**	**Sex**		
	
	Male	1.00	1.00
	
	Female	0.95 (0.87-1.04)	1.07 (1.02-1.13)
	
	**Age**		
	
	< 18	0.03 (0.01-0.06)	0.46 (0.39-0.54)
	
	18-24	0.69 (0.47-1.01)	0.68 (0.54-0.85)
	
	25-34	0.58 (0.41-0.80)	0.76 (0.64-0.91)
	
	35-44	1.00	1.00
	
	45-54	1.07 (0.86-1.34)	1.27 (1.12-1.43)
	
	55-64	1.30 (1.05-1.59)	1.70(1.51-1.92)
	
	65-74	1.68 (1.38-2.04)	2.79 (2.49-3.12)
	
	75+	2.21 (1.83-2.68)	6.22 (5.58-6.94)

**Need Factors**	**Charlson Comorbidity Index**		
	
	0-1	1.00	1.00
	
	2-3	0.93 (0.84-1.03)	1.29 (1.21-1.37)
	
	≥ 4	0.43 (0.38-0.49)	1.71 (1.61-1.82)
	
	**Length of Stay (Percentile)**		
	
	< 25^th^	1.00	1.00
	
	25-50^th^	3.16 (2.50-4.00)	0.55 (0.51-0.59)
	
	50-75^th^	7.80 (6.26-9.73)	0.68 (0.64-0.73)
	
	75-90^th^	16.18 (12.89-20.30)	1.17 (1.08-1.27)
	
	90^th^+	23.07 (18.12-29.37)	1.92 (1.73-2.12)
	
	**Special Care Days**		
	
	None	1.00	1.00
	
	1-2	1.30 (1.13-1.50)	1.19 (1.10-1.28)
	
	3-5	1.71 (1.46-2.00)	2.24 (2.05-2.46)
	
	6-11	2.20 (1.84-2.63)	3.63 (3.25-4.11)
	
	12+	3.02 (2.51-3.63)	4.10 (3.58-4.69)

**Enabling Factors**	**Rural**		
	
	No	1.00	1.00
	
	Yes	0.78 (0.68-0.88)	1.07 (1.00-1.14)

Finally, all predisposing, need, and enabling variables were significant predictors of discharge to other institutionalized care compared to home among nTBI patients. Specifically, females (OR = 1.07) and those living in a rural area (OR = 1.07) were significantly more likely to be discharged to 'other' and there was a linear relationship with the odds of discharge to 'other' with age, Charlson Comorbidity Index score, length of stay, and number of special care days (see Table 5).

## Discussion

To our knowledge, this is the first paper that models outcomes from hospital admissions for both TBI and nTBI from a population based perspective in a publicly insured setting. Using a comprehensive database of hospitalization in Ontario, this paper demonstrates that the need, predisposing, and enabling factors are significant predictors to inpatient rehabilitation or institutionalized care compared to home settings among the ABI population. Our results also corroborate with findings from previously published studies, which also found that patients with longer lengths of stay and older age to be significantly more likely to be discharged to inpatient rehabilitation and 'other' and that females were significantly more likely to be discharged to 'other'. The strength of this study is that we have captured information across an entire population and as such, it is highly generalizable. The statistical modeling also allowed us to compare the contribution of covariates on multiple outcomes. Furthermore, Ontario's Discharge Abstract Database has the advantage of covering all hospitalizations and the ABI Dataset Project uses a definition that covers mild brain injuries and nTBI as well.

However, findings must be interpreted keeping in mind the limitations of the study. Firstly, we recognize the inherent limitation of conducting research using administrative data [14]. Our data were based on discharge destination data from acute care and not based on actual linking of records across the continuum. As a result, there is the possibility of misclassification bias. In addition, we identified the cohort based on ICD-10 coding regardless of severity or whether the brain injury was the most responsible diagnosis category. As a result, the cohort identified will be quite diverse in terms of their brain injury. Also, we did not have a direct measure of the initial severity of the brain injury for all patients in the administrative data. Although the GCS is available, it is not a variable that is mandatory for collection in the administrative datasets used for this study and thus, inclusion of the GCS would have resulted in reducing of large portion of the sample, as GCS was not available in over 40% of the ABI episodes (data not shown). However, it should be noted that the GCS is not a strong predictor of longer-term functional status [15]. In addition, we were not able to measure variables such as ethnicity as it is not available in administrative databases. We also recognize that discharge destinations such as residential care is influenced by the availability of a caregiver and other supports, which is also not available in the administrative data.

Nonetheless, our results demonstrate that with increasing age, the odds of discharge to inpatient rehabilitation and to other institutionalized care significantly increased. In particular, older adults aged 65 years and older were 11% to 522% more likely to be discharged to inpatient rehabilitation or to institutionalized care compared to the middle age group (35 - 44 years). Given that older adults are the fastest growing segment of the population, policy makers need to prepare for a significant potential need these services in the near future.

Furthermore, increasing length of stay significantly increased the odds of discharge to non-home settings. For example, TBI patients with a length of stay in the 90^th ^percentile were 160.83 times as likely as those in the 25^th ^percentile to be discharged to inpatient rehabilitation and 4.85 times as likely to be discharged to 'other'. Similarly, TBI patients with 12 or more special care days were 4.55 times as likely to be discharged to inpatient rehabilitation and 3.49 times as likely to be discharged to 'other' compared to those without any special care days. Similar trends were observed among nTBI patients. These factors should be used to better guide discharge planning in the acute care setting and suggests that those with more severe injuries are more likely to be discharged somewhere other than home.

In addition, we found that MVC is a significant predictor of discharge to inpatient rehabilitation among TBI patients, such that these patients were 1.49 as likely as those who were not involved in a MVC to be discharged to this destination. Previous work in Ontario has shown that persons injured through MVC were less likely to wait for inpatient rehabilitation services controlling for factors such as age and severity of injury [16]. Recently there has been a reduction in the amount of MVC coverage in the province of Ontario for post acute care. The impact of this reduction needs to be monitored on health service utilization after acute care.

Of particular importance is our finding that ABI patients living in rural areas were approximately 25% less likely to be discharged to inpatient rehabilitation. Previous studies have identified that geographic distance and boundaries are obstacles in providing and accessing ABI services [17]. Thus, there is an increased need for attention in creating a more supportive environment at home for these patients. For example, Bergquist et al. examined the effects of an internet-based cognitive rehabilitation program for individuals with memory impairments after severe TBI. Using an instant messaging system over the internet, participants interacted with a therapist on the internet and were taught to use a calendar and a diary to compensate for memory problems. Results showed evidence for improved memory and mood following completion of all sessions and suggest that internet based cognitive rehabilitation may be beneficial [18]. Also, Arundine and colleagues incorporated telephone cognitive behavioural therapy (CBT) in their assessment of psychological distress among ABI patients and found commensurate benefits of telephone CBT relative to conventional treatment [19]. Thus, it suggests that innovations in health care delivery can make some impact in barriers to health care services. Funding models need to address these innovations.

## Conclusions

Our findings show that need factors are most associated with discharge destination, however, the availability of supplemental insurance and geography also influence services. It also provides a profile of the characteristics of traumatic and non traumatic brain injury. In planning services for those with ABI, geographical access should be taken into account with rural areas in Ontario to be targets of increased access to care. This can be potentially accomplished through innovations in reducing the barriers to ABI services. In addition, findings that private insurance is a factor in access to services, even in a publicly funded healthcare system, implies that better planning should be in place to ensure more equitable access to appropriate treatment and services among persons with ABI.

Future research should examine additional factors that may be associated with discharge destination among ABI patients in acute care. Factors of interest that were not available in this study include social support, living situation prior to ABI and Glasgow Coma Scale on admission. Psychosocial factors have also been raised as important factors in access to services including attitude and knowledge of services, social norms, and perceived control. Finally, there are limited indicators from acute care that address functional status on discharge and thus, mandatory collection of more relevant indicators on discharge should be considered. Ultimately, research on these characteristics and factors should be used to inform policy and programs to ensure the best outcomes for patients with ABI after discharge from acute care.

## Competing interests

The authors declare that they have no competing interests.

## Authors' contributions

DP, RV BZ, and AC conceptualized the study. BZ formulated the methods for statistical analysis and carried out the statistical analysis using SAS software. AYC drafted the paper, conducted a literature review and interpreted the results that formulated the foundation of the paper. VC, DP, AC, RV, and BZ all had significant input into the editing process of the paper and additional interpretation of results. All authors read and approved the final manuscript.

## Pre-publication history

The pre-publication history for this paper can be accessed here:

http://www.biomedcentral.com/1471-2377/12/16/prepub
